# UC2288 induces cell apoptosis of nasopharyngeal carcinoma cells via inhibiting EGFR/ERK pathway

**DOI:** 10.7150/jca.48282

**Published:** 2021-01-01

**Authors:** Renba Liang, Xiaodong Zhu

**Affiliations:** 1 Department of Oncology, Wuming Hospital of Guangxi Medical University, Nanning, Guangxi, P.R. China.; 2 Department of Radiation Oncology, Guangxi Medical University Cancer Hospital, Nanning, Guangxi, P.R. China.; 3 Key Laboratory of Early Prevention and Treatment for Regional High‐Incidence‐Tumor, Guangxi Medical University, Ministry of Education, Nanning, Guangxi, P.R. China.

**Keywords:** nasopharyngeal carcinoma, UC2288, proliferation, apoptosis, EGFR/ERK pathway

## Abstract

Radiotherapy and chemotherapy are the standard care for patients with nasopharyngeal carcinoma (NPC). These treatments cause some severe toxicity and about 30% of patients develop recurrence and metastases after treatment. UC2288 is structurally similar to sorafenib, a multikinase inhibitor. However, studies about the effects of UC2288 on tumors are few. Here, UC2288 inhibited proliferation and induced apoptosis of NPC cells in a dose- and time-dependent manner. Using western blot and immunofluorescence assay, we found that UC2288 promoted DNA damage. In addition, UC2288 decreased the phosphorylation of EGFR and ERK. Moreover, pretreatment with EGF partially rescued cell viability suppressed by UC2288. In conclusion, UC2288 suppressed the growth of NPC via inhibiting EGFR/ERK pathway and it may be a promising therapeutic option for NPC.

## Introduction

Nasopharyngeal carcinoma (NPC) is a malignant epithelial tumor from the nasopharynx, which occurs mostly in southern China and Southeast Asia 1. The standard treatment for NPC is intensity-modulated radiotherapy (IMRT) and platinum-based chemotherapy 1. With great advance having made in the treatment in the contemporary era, the survival rate of most patients has greatly improved. However, 20~30% of patients still develop relapses and metastases after system treatment 1, 2. Those patients with treatment failure receiving re-irradiation have severe side-effects, such as xerostomia, mucosal necrosis and temporal lobe necrosis 3. As a first-line therapy in recurrent or metastatic NPC, platinum-based chemotherapy response doesn't last long. Therefore, it is necessary to explore more therapeutic strategies to provide new options for the treatment of NPC.

**Figure 1:**
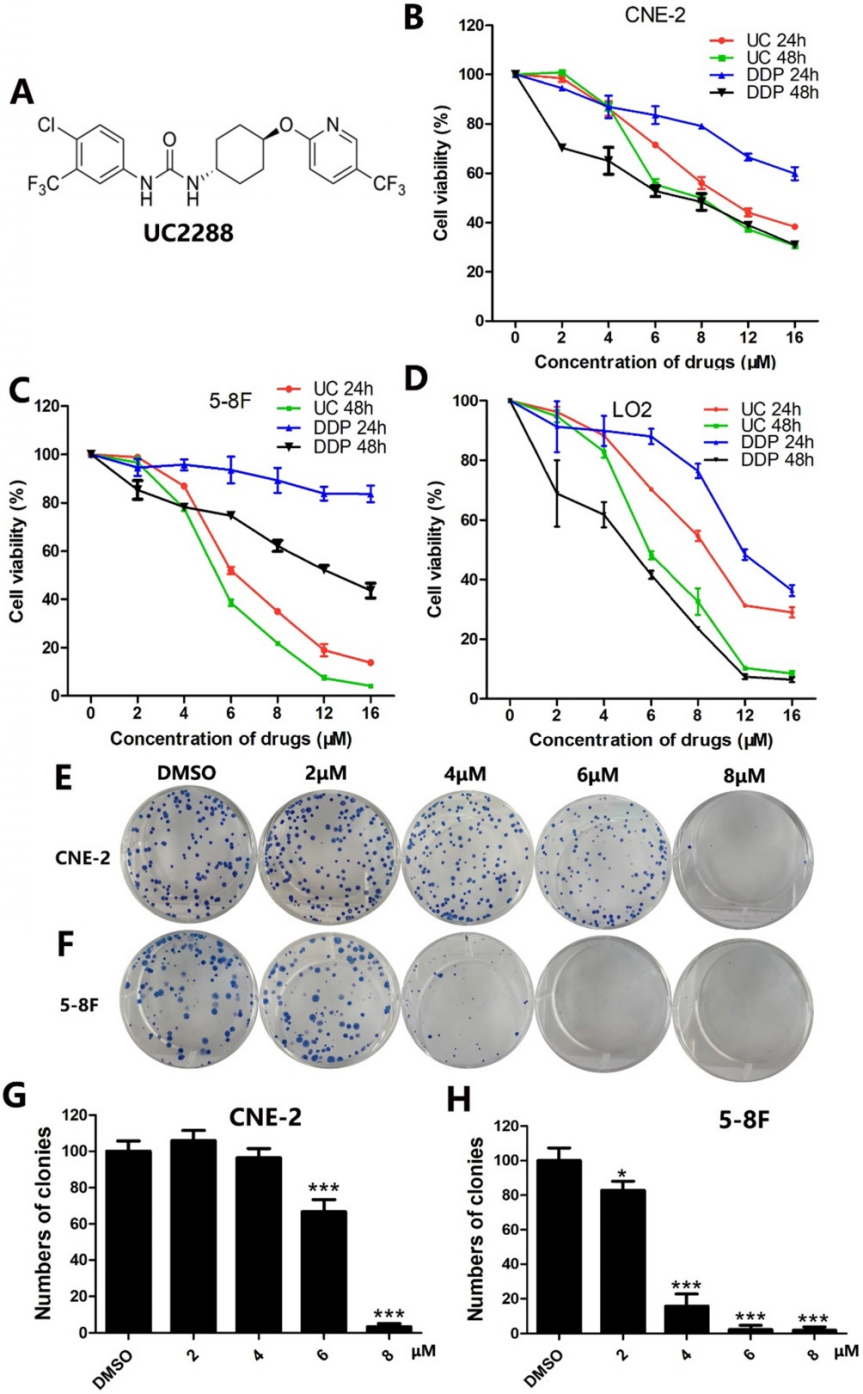
** UC2288 decreased proliferation of NPC cells.** (A) The chemical structure of UC2288. (B-D) NPC cells and human immortalized hepatocytes L-02 were treated with UC2288 or DDP at indicated concentrations for 24 and 48 h, respectively. Cell viability was detected by CCK8 assay. (E-H) After treatment with UC2288 for 48 h and then incubation with medium for 12 days, the colony formation assay was conducted. Data are expressed as mean ± SD, n=3. *p < 0.05 and ***p < 0.001 compared to control.

UC2288 is called trans-1-(4-chloro-3-trifluoromethyl-phenyl)-3-[4-(5-trifluoromethyl-pyridin-2-yloxy)-cyclohexyl]-urea 4 (Fig. 1A). The structure of UC2288 is similar to that of sorafenib, a multikinase inhibitor 4. However, unlike sorafenib, UC2288 has minimal inhibitory properties on vascular endothelial growth factor receptor 2 (VEGFR2) and Raf kinase activity as evidenced by the finding that changes of p-ERK levels varied in renal cell carcinoma cell lines and a normal kidney cell treated with UC2288 4. So far, there are very few reports on the role of UC2288 in tumors. Whether UC2288 has any effects on NPC is unclear. Herein, we revealed that UC2288 suppressed proliferation and induced apoptosis in NPC cells. Moreover, inhibition of epidermal growth factor receptor/extracellular signal-regulated kinase (EGFR/ERK) signaling is vital for UC2288-mediated anticancer activity.

## Materials and methods

### Chemicals and reagents

UC2288 (purity ~99%) and human recombinant epidermal growth factor (EGF) were purchased from Abcam (Cambridge, UK), and dissolved in dimethyl sulfoxide (DMSO) and water, respectively. Cisplatin (Haosen, Jiangsu, China) was dissolved in water. The primary antibodies including EGFR, p-EGFR (Tyr1068), ERK1/2, p-ERK1/2 (Thr202/Tyr204), caspase 3, cleaved-caspase 3, γ-H2AX, survivin, Bax, Bcl-2, GAPDH and goat anti-rabbit IgG-HRP and Alexa Fluor-488-labeled goat anti-rabbit (IgG) secondary antibody secondary antibodies were obtained from Cell Signaling Technology (Massachusetts, USA).

### Cell culture

The human nasopharyngeal carcinoma cell lines CNE-2 and 5-8F and human immortalized hepatocytes L-02 were used in the study. CNE-2 was purchased from Cancer Hospital of Fudan University (Shanghai, China) and tested through DNA (STR) profiling on 15 March 2018. 5-8F was obtained from American Type Culture Collection (Rockville, MD, USA). L-02 cells were purchased from Procell company (Wuhan, China). The cells were cultured in Dulbecco's modified Eagle's medium (DMEM) containing 10% fetal bovine serum (FBS), 100 U/mL penicillin, and 100 µg/mL streptomycin (Gibco; Thermo Fisher Scientific, USA) at 37°C with 5% CO2. Experiments were performed when cells grew to 80% and there were no more than 20 passages of cells in the experiments.

### Cell viability assay

To evaluate the anti-proliferation of UC2288 on cells, CCK-8 assay was performed. Briefly, cells were seeded in 96-well plates at a density of 3500 cells per well and treated with various concentrations of UC2288 for 24 h or 48 h. Negative control group was treated with medium containing the same volume of DMSO. Then, 10 μL CCK8 was added in per well and incubated at 37°C in dark for 2 hours. The optical density (OD) value was measured at 450 nm using a spectrophotometer (BioTek Instruments, Inc., USA).

### Colony formation assay

The long-term inhibitory effect of UC2288 on cells was assessed by colony formation assay. Cells (300 cells/well) were seeded in 6-well plates and cultured overnight. Then, the cells were treated with various concentrations of UC2288 for 48 h and discarded the drugs, and cultured in fresh medium for 12 days. After that, cells were washed with phosphate-buffered saline (PBS) twice, fixed with 4% paraformaldehyde for 30 min and stained with Giemsa Stain solution (Beyotime, China) for 30 min. The number of colonies was counted after the plates were dried.

### Hematoxylin-Eosin staining

To examine the morphologic changes of cells, Hematoxylin-Eosin staining was conducted. Cells were cultured on coverslips placed in 6-well plates and treated with indicated doses of UC2288 for 24 h. Then cells were washed with PBS three times, fixed with 95% ethanol for 20 mins, and washed with PBS twice. After that, cells were stained with hematoxylin for 3 min, washed with water, dyed with eosin for 1 min and wash with water. After drying, cells were detected under microscope (Life technologies, USA).

### Apoptosis assay

Annexin-V-PE/7-AAD double staining was conducted to determine the effect of UC2288 on cell apoptosis. Cells were seeded in 6-well plates and cultured for 24 h. Then, the cells were treated with various concentrations of UC2288 or vehicle (DMSO) for 48 h. Next, cells were collected and stained with apoptosis detection kit (BD, Biosciences, USA) according to the manufacturer's protocol. The apoptosis cells were evaluated by a FACSCalibur™ flow cytometer (BD Biosciences, USA).

### Immunofluorescence assay

Cells were cultivated on coverslips placed in 6-well plates and treated with different concentrations of UC2288 for 24 h. The samples were fixed with 4% paraformaldehyde for 20 mins, washed with PBS twice, and blocked with immunol staining blocking buffer (Beyotime, China) for 1 h. The cells were stained with γ-H2AX at 4°C overnight and Alexa Fluor-488-labeled goat anti-rabbit (IgG) secondary antibody for 1 h in dark at room temperature and DAPI for 15 min in dark. Finally, cells were detected under fluorescence microscope (Life technologies, USA). The γ-H2AX foci were calculated by Image-Pro Plus (Media Cybernetics). At least 50 cells in each group were used for quantitative analysis of foci formation.

### Western blot analysis

Cells were cultured in 6-well plates and treated with different concentrations of UC2288 for 24 h. Whole cell protein extracts were obtained using RIPA buffer which contained proteinase inhibitors (1 mM PMSF). The protein samples were separated through 6-10% SDS-PAGE and sequentially transferred to polyvinylidene fluoride (PVDF) membranes. Then, the membranes were blocked with 5% nonfat milk for 1 h and incubated with primary antibodies at 4°C overnight. After that, membranes were washed with TBS-Tween three times (5 mins/per time) and incubated with a peroxidase-conjugated secondary antibody for 1 h at room temperature and washed with TBS-Tween three times again. Signals were detected using ECL chemiluminescent enhanced reagent under a Bio-Rad System (Hercules, California, USA) and quantified by ImageJ software.

### Statistical analysis

All experiments were conducted at least three times independently. The data were reported as mean ± standard deviation (SD) and analyzed by Student's t-test or one-way analysis of variance (ANOVA) using the Graphpad software (La Jolla, CA, USA). The difference between groups was considered statistically significant when *p*-value < 0.05.

## Results

### Inhibitory Effects of UC2288 on NPC cell proliferation

To evaluate whether UC2288 could inhibit the proliferation of NPC cells, cell viability was assessed by CCK-8 assay. The data demonstrated that UC2288 dramatically suppressed cell viability in a time- and dose-dependent manner. Of particular note is that the cell viability of CNE-2 dropped to 86.88% (4 μM), 55.41% (6 μM), 49.93% (8 μM) and 37.06% (12 μM) and correspondingly 77.68% (4 μM), 38.55 (6 μM), 21.82% (8 μM) and 7.41% (12 μM) in 5-8F cells compared with control after treatment of UC2288 for 48 h (Fig. 1B, C). The IC50 (half maximal inhibitory concentration) at 24 h was 10.83 μM and 6.95 μM in CNE-2 and 5-8F, respectively. It is generally accepted that cisplatin (DDP) is a well-known component of neoadjuvant chemotherapy or adjuvant chemotherapy for NPC. However, UC2288 had a stronger inhibitory effect on 5-8F cells than DDP (Fig. 1C). Moreover, the cytotoxicity of UC2288 on human immortalized hepatocytes L-02 was lower than that of DDP at 48h time point (Fig. 1D). In addition, UC2288 significantly reduced the clonogenicity of NPC cells (Fig. 1E-H). Collectively, these findings unveiled that UC2288 was endowed with anti-proliferation property in NPC.

### UC2288 triggers apoptosis of NPC cells

**Figure 2:**
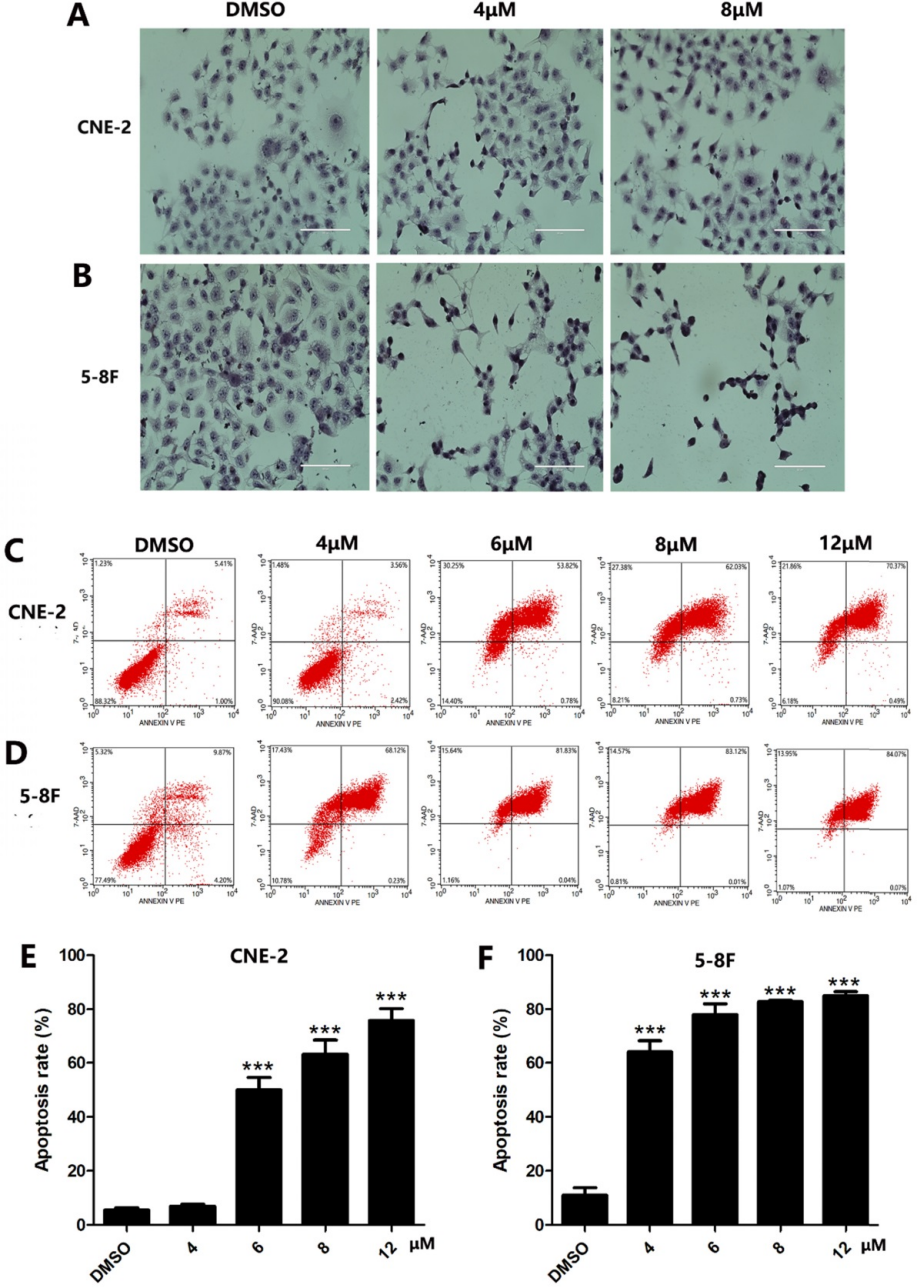
** The morphologic changes and apoptosis induced by UC2288.** (**A-B**) Cells were treated with indicated concentrations of UC2288 for 24 h and then H&E stain was performed to observe morphologic changes of cells (scale bar=100 µm). (**C-D**) After treatment with UC2288 for 48 h, cells were stained with Annexin-V-PE/7-ADD and were evaluated by flow cytometry. (**E-F**) Quantification of apoptotic cells. Data are expressed as mean ± SD, n=3. ***p < 0.001 compared to control.

**Figure 3:**
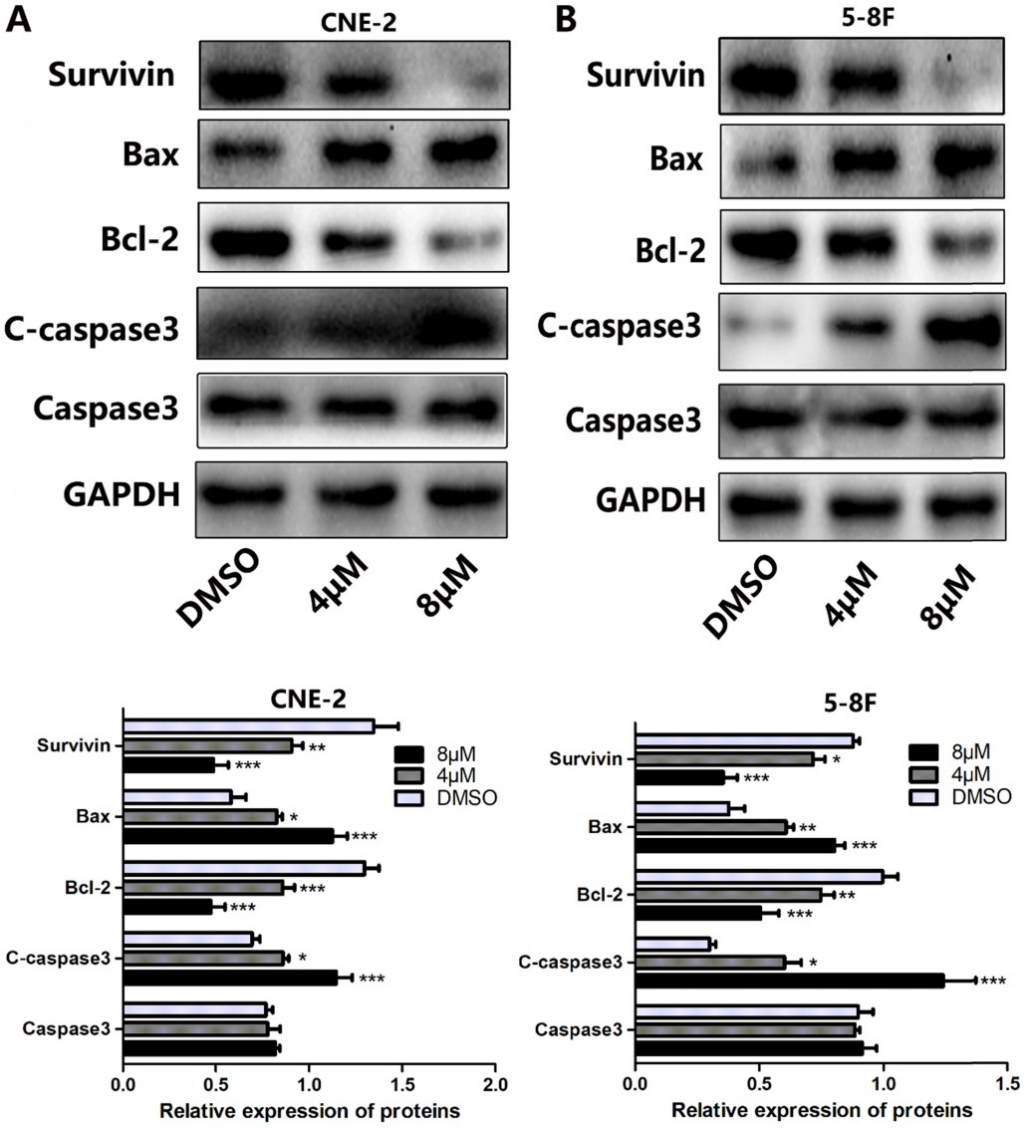
** The effects of UC2288 on apoptosis-related proteins.** (**A-B**) The expression levels of apoptosis-related proteins were measured by western blot (A. CNE-2. B. 5-8F). Data are expressed as mean± SD, n=3. *p < 0.05, **p < 0.01 and ***p < 0.001 compared to control.

Since apoptosis is a crucial index to analyze cell growth, we then raise a question as to whether UC2288 induces apoptosis in NPC cells. After 48 h incubation of UC2288, there were significant changes in cell morphology, including cytoplasm reduction, nucleus shrinkage, and chromatin condensation (Fig. 2A, B). Furthermore, Annexin V-PE/7-ADD staining indicated that UC2288 (0-12 μM) concentration-dependently gave rise to increase of apoptotic cells, ranging from 5.37% to 75.67% in CNE-2 and from 10.95% to 84.95% in 5-8F cells (Fig. 2C-F). In addition, as shown by Western Blot, UC2288 significantly increased cleaved-caspase3 and Bax expression, accompanied by a parallel decreased Bcl-2 and survivin expression (Fig. 3A, B). These data further corroborated the findings of apoptosis induced by UC2288.

### UC2288 induces DNA damage in NPC cells

**Figure 4:**
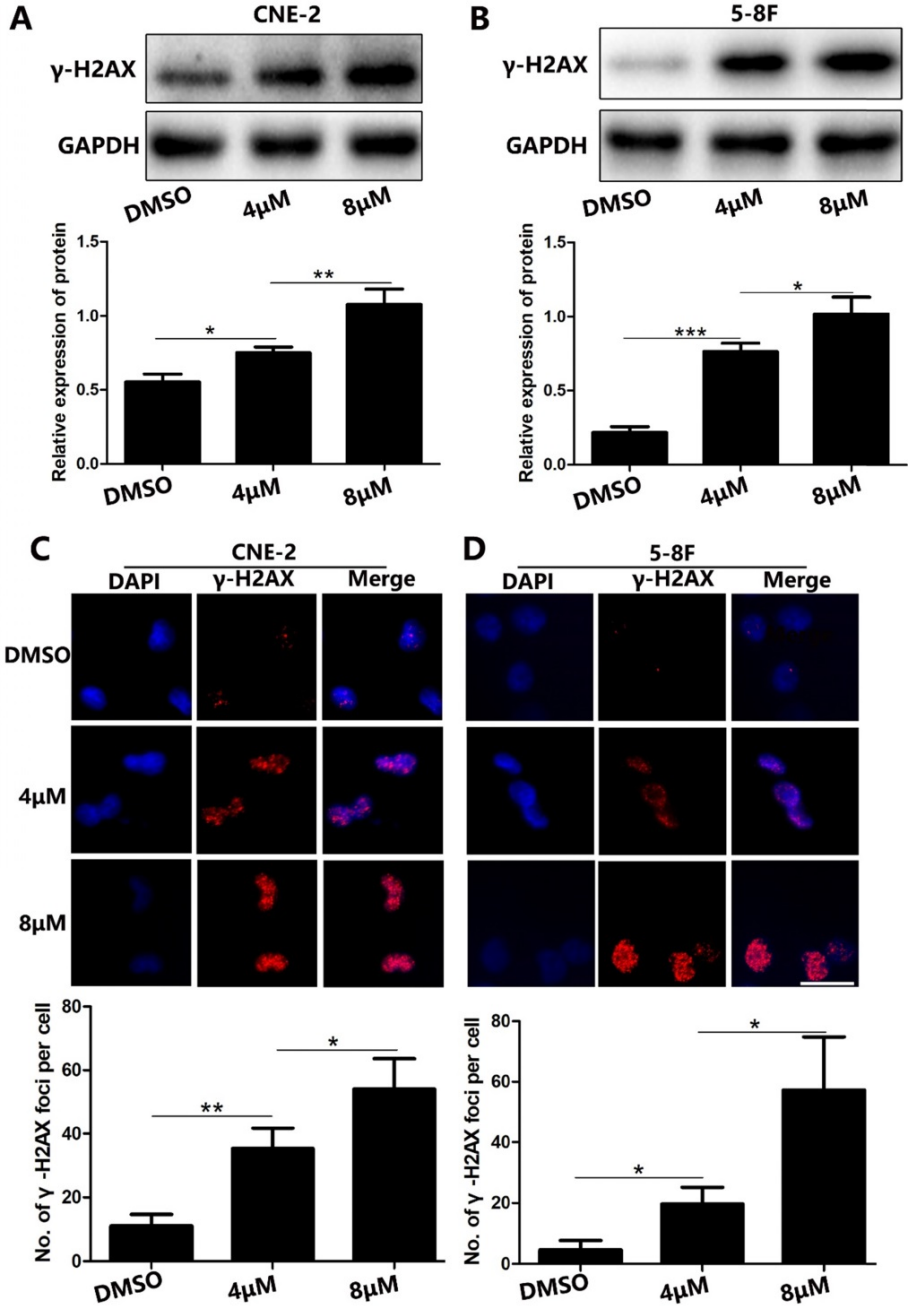
** UC2288 induced DNA damage in NPC cells.** (**A-B**) The γ-H2AX expression was measured by western blot. (**C-D**) The γ-H2AX foci were detected under fluorescence microscope. Data are expressed as mean ± SD, n=3. *p < 0.05, **p < 0.01 and ***p < 0.001. Scale bar = 20 µm.

Because DNA damage is an important characteristic of cell apoptosis 5, 6, we examined whether UC2288 induced DNA damage. We found that γ-H2AX, a sensitive biological marker for DNA double-strand breaks (DSBs) 7, was upregulated in UC2288-treated cells (Fig. 4A, B). We also conducted immunofluorescence assay to evaluate the expression of γ-H2AX qualitatively and quantitatively. In concordance with the findings of western blot, we observed the number of γ-H2AX foci significantly increased after the treatment of UC2288 (Fig. 4C, D). These results suggested that UC2288 promoted DSBs.

### UC2288 inhibits EGFR/ERK signaling pathway in NPC cells

**Figure 5:**
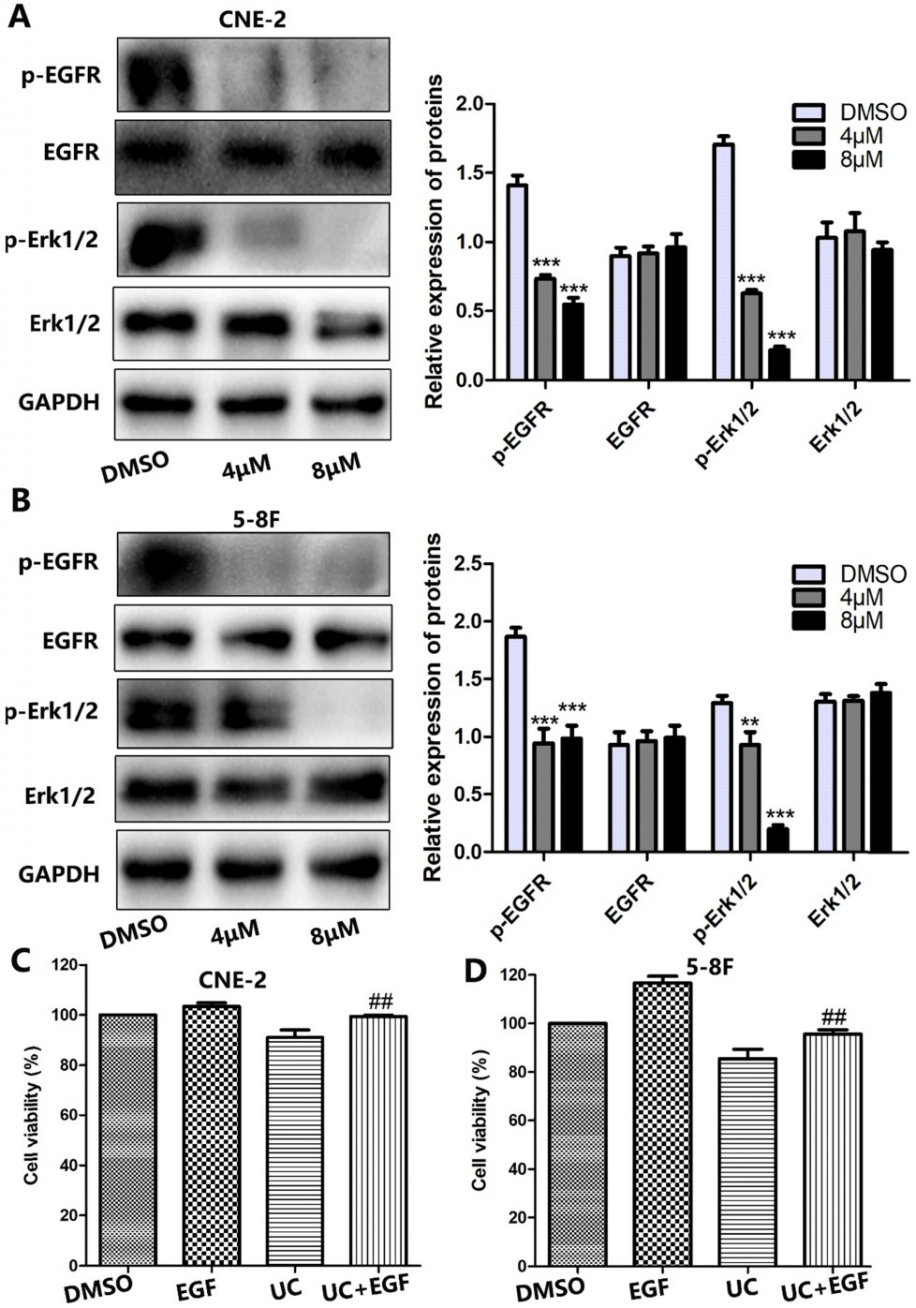
** UC2288 inhibited EGFR/ERK pathway.** (**A-B**) The expression of p-EGFR/EGFR and p-ERK1/2/ERK was examined by western blot. Data are expressed as mean ± SD, n=3. **p < 0.01 and ***p < 0.001 compared to control. (**C-D**) Pretreatment with epidermal growth factor (EGF), CCK-8 assay was conducted to evaluate the cell viability. Data are expressed as mean ± SD, n=3. **^##^***p* < 0.01 compared to the UC2288 (4 µM)-treated group.

Growing evidence has indicated that the majority of NPCs express high levels of EGFR 8. Thus, this leads us to question whether EGFR signaling is involved in UC2288-induced NPC growth inhibition. To test our hypothesis, we first performed western blot. As shown by Figure 5A, B, UC2288 reduced the expression level of phosphorylation of EGFR (p-EGFR) and phosphorylation of ERK1/2 (p-ERK1/2). However, there was no significant change on the expression of EGFR and ERK1/2 after UC2288 treatment. Epidermal growth factor (EGF) can bind to EGFR and activate the downstream molecules of EGFR signaling. Next, EGF was used to further probe the role of EGFR/ERK pathway in UC2288 anti-cancer ability. We found that UC2288 decreased cell viability, while EGF partially abrogated UC2288-induced NPC cells suppression (Fig. 5C, D). Detailedly, cell viability increased from 91.04% in the UC2288 group to 99.40% in the EGF+UC2288 group and from 85.43% to 95.52% in CNE-2 and 5-8F, respectively. Taken together, these results suggested that UC2288 inhibited NPC growth via repressing EGFR/ERK signaling pathway.

## Discussion

Proliferation is one of the vital characteristics of tumors which contributes to cancer development and progression 9. In light of the important role in tumors, targeting proliferation is a promising therapeutic strategy. In the present study, we found that UC2288 inhibited proliferation of NPC cells in a dose- and time-dependent manner. Moreover, the results showed that UC2288 had higher cytotoxicity in 5-8F cells than cisplatin, which is widely used in chemotherapy for nasopharyngeal carcinoma 10. In addition, UC2288 also suppressed colony-formation ability in NPC cells, indicating that the drug exerted an inhibitory effect on the long-term growth of cells. These results were in agreement with the findings of previous study that UC2288 inhibited cell growth of other cancer cells 4.

Apoptosis is another key factor responsible for tumors development 11, 12. Many chemotherapy agents display anti-tumor effects by inducing apoptosis 13. In terms of morphological changes, apoptosis cells undergo cell shrinkage and nuclear condensation 14. We then examined the apoptosis induced by UC2288 with different approaches. As predicted, UC2288 resulted in morphological changes in NPC cells as discussed above. Furthermore, the pro-apoptosis property of UC2288 was further corroborated by the Annexin V-PE/7-AAD assay. Growing evidence has demonstrated that there are two major pathways contributing to apoptosis, extrinsic (death receptor) pathway and intrinsic (mitochondria) pathway 15. Death receptor can activate caspases, which subsequently trigger cell apoptosis 16. With regard to intrinsic pathway, cytochrome c and Bcl-2 family proteins (Bcl-2, an anti-apoptotic protein; Bax, a pro-apoptotic protein) are pivotal for it 17. Moreover, these two apoptotic pathways affect each other, as caspases mediate Bax and Bax enhances cytochrome c release from mitochondria and caspases activation 16, 18. Our results revealed that treatment with UC2288 gave rise to upregulation of cleaved-caspase3 and Bax and reduction of Bcl-2. This finding suggested UC2288 may trigger NPC cells apoptosis through extrinsic pathway and intrinsic pathway.

DNA is one of the main targets of many chemotherapy agents and stress 19 and DNA damage commonly occurs in these genotoxic agents-induced apoptosis 5. It is particular to note that DNA double-strand breaks (DSBs) is the most severe DNA damage. Interestingly, we found that UC22888 stimulated the formation of DSBs as demonstrated by the upregulation of γ-H2AX in western blot and γ-H2AX foci in immunofluorescence assay. This finding further confirmed the pro-apoptosis ability of UC2288.

EGFR is upregulated in most NPC samples and combination anti-EGFR antibody with chemotherapy is an effective, promising and well-tolerated strategy for recurrent or metastatic NPC 20, 21. Extracellular-signal regulated kinase (ERK) is one of downstream molecules of EGFR signaling. Evidence has demonstrated that ERK stimulates cancer cell proliferation 22, 23 and suppresses apoptosis 24, 25. Moreover, EGFR/ERK pathway plays a vital role in nasopharyngeal carcinoma progress 26. It was reported that UC2288 exerted inhibitory effects on cell viability through suppressing p21 4. However, since sorafenib is proved to be an ERK signaling inhibitor and the structure of UC2288 is similar to that of sorafenib, we thus asked whether EGFR/ERK pathway was involved in UC2288 anti-cancer activity. Our data revealed that UC2288 inhibited the activation of EGFR and ERK. Furthermore, EGF partly rescued cell viability suppressed by UC2288. Collectively, these results suggested that EGFR/ERK pathway was involved in UC2288-induced growth inhibition in NPC.

In summary, this study showed that UC2288 decreased cell proliferation and induced apoptosis in nasopharyngeal carcinoma cells through inhibition of EGFR/ERK pathway. Our results indicated that UC2288 may be a promising and effective anti-cancer agent.
